# Single-Cell Atlas of Human Temporomandibular Joint Condyle Identifies *MXD4* as a Regulator of Chondrogenesis

**DOI:** 10.1016/j.identj.2026.111096

**Published:** 2026-09-03

**Authors:** Liang Wang, Yiyi Chen, Yuxin Zhang, Nan Zhao, Teng Xu, Xinmei Liu, Jisi Zheng, Dong Liu, Xingmei Feng

**Affiliations:** a Department of Stomatology, Affiliated Hospital of Nantong University, Institute of Special Environmental Medicine, Nantong University, Nantong, China; b Department of Stomatology, Affiliated Hospital of Nantong University, Medical School of Nantong University, Nantong, China; c Department of Oral Surgery, Shanghai Ninth People’s Hospital, Shanghai Jiao Tong University School of Medicine, College of Stomatology, Shanghai Jiao Tong University, National Center for Stomatology, National Clinical Research Center for Oral Diseases, Shanghai Key Laboratory of Stomatology, Shanghai Research Institute of Stomatology, Research Unit of Oral and Maxillofacial Regenerative Medicine, Chinese Academy of Medical Sciences, Shanghai, China; d School of Life Science, Nantong Laboratory of Development and Diseases, and Co-Innovation Center of Neuroregeneration, Nantong University, Nantong, China

**Keywords:** Chondrocytes, Embryonic development, Endochondral ossification, RNA sequencing, Transcription factors, Zebrafish

## Abstract

**Introduction and aims:**

The temporomandibular joint condyle (TMJC) is essential for mandibular and temporomandibular joint (TMJ) development. However, its cellular and molecular dynamics during human embryogenesis remain poorly understood.

**Methods:**

We constructed a single-cell transcriptomic atlas of the human TMJC spanning five embryonic stages from 12 to 25 gestational weeks (GW12-25). Single-cell RNA sequencing was performed to identify cell types, and Monocle 3 and SCENIC analyses were used to infer developmental trajectories and identify key transcriptional regulators. Candidate regulators were functionally validated using zebrafish models.

**Results:**

Single-cell RNA sequencing identified 20 major cell types within the TMJC. Immune cells were sparse at GW19 but expanded markedly by GW22. Trajectory analysis revealed a differentiation pathway from mesenchymal stem cells to chondrocytes. SCENIC analysis highlighted multiple transcription factors regulating distinct cell populations, among which *MXD4* was identified for the first time as a candidate transcriptional regulator of cartilage development. Functional studies showed that *mxd4* knockout in zebrafish led to craniofacial developmental abnormalities.

**Conclusion:**

This single-cell atlas provides a comprehensive resource for understanding the cellular and molecular mechanisms underlying TMJC development. *MXD4* is identified as a candidate regulator in condylar morphogenesis.

**Clinical relevance:**

Preliminary investigation of the potential regulatory role of *MXD4* in condylar cartilage formation may provide a theoretical basis for diagnostic, therapeutic, and translational studies of craniofacial malformations associated with condylar hypoplasia.

## Introduction

The temporomandibular joint (TMJ) is a synovial joint primarily composed of the mandibular condyle, the articular surface of the temporal bone, the articular disc, the joint capsule, and associated ligaments.^1^, ^2^, ^3^ The TMJ condyle (TMJC), a crucial component of the TMJ, is predominantly covered by condylar cartilage and subchondral bone, and plays a vital role in mandible growth in both length and height.^4^, ^5^, ^6^ This growth is driven by the endochondral osteogenesis of the mandibular condylar cartilage (MCC).^5^^,^^7^^,^^8^ MCC dysplasia can lead to abnormal condylar morphology, potentially affecting mandibular and facial development. This can significantly affect patients’ physical and mental well-being.^9^, ^10^, ^11^

Unlike other articular cartilages, MCC is classified as secondary cartilage.^5^^,^^7^^,^^12^ Undifferentiated cells in the inner layer of the perichondrium continuously differentiate and proliferate towards the cartilage surface, forming mature chondrocytes. These chondrocytes expand outward from the surface and eventually mineralize to form new bone.^13^^,^^14^ Additionally, the transformation of chondrocytes into osteocytes during condylar development is closely linked to the surrounding bone marrow environment. Mesenchymal stem cells (MSC) and hematopoietic stem cells in the bone marrow play essential roles in the formation and remodelling of bone tissue. Particularly during condylar development, these cells support bone growth and development.^4^^,^^15^ The formation of the TMJ involves multiple cell types, signalling pathways, and local microenvironmental factors; however, its molecular mechanisms remain complex and incompletely understood. Therefore, studying the development of human embryonic condylar tissue is of significant importance.

Numerous studies have investigated the development of the TMJ. For instance, Lee et al^16^ examined the continuous growth patterns of the mandible in 38 embryos and 111 foetuses using serial histological sections and soft X-ray examinations, revealing the cone-shaped structure and abundant bone marrow hematopoietic tissue in condylar growth. Molina^17^ conducted histochemical studies on the mandibular condyle of foetuses at 12, 14, and 16 gestational weeks (GW12, GW14, and GW16). They discovered that endochondral bone formation in the mandibular condyle involves changes in glycoprotein levels, mesenchymal tissue, and collagen. Although these histological and histochemical studies have provided important anatomical and developmental information, they do not resolve the cellular heterogeneity and gene regulatory programs that drive human TMJC development at single-cell resolution.

In our previous research, we first employed single-cell RNA sequencing (scRNA-seq) to analyse the condylar tissue of 3- and 4-month-old human embryos, constructing a single-cell atlas of condylar development.^2^ However, condylar development extends beyond the third and fourth months of embryonic development. Previous studies have divided the development of the TMJ in human embryos into four key stages: (1) Preparation stage (before GW8 of embryonic development): At this stage, a true TMJ has not yet formed, and the malleus-incus joint performs the original TMJ function. (2) Initial stage (GW8-9): The temporal and condylar anlagen appear, and the condylar anlage begins endochondral ossification. (3) Differentiation stage (GW10-20): During this stage, the articular disc, joint cavity, and synovium are formed, secondary cartilage appears, and the condyle undergoes further endochondral ossification. (4) Completion stage (GW20 to birth): At this final stage, the various components of the TMJ are fully formed, Meckel’s cartilage disappears, the articular disc undergoes further remodelling, the condyle continues endochondral osteogenesis, intramembranous ossification occurs in the fossa, and hematopoietic tissue emerges.^2^^,^^5^ Therefore, we continue to sequence condylar tissues at GW12, GW22, and GW25 to further refine the human condylar developmental atlas. This research aims to contribute to the development of innovative targeted and biological therapies for disorders related to TMJ development.

In this study, we constructed a comprehensive single-cell transcriptomic atlas of human embryonic TMJC tissues across five developmental stages. We further screened transcription factors related to cell subtypes and developmental trajectories during condylar development, and identified *MXD4* as a candidate transcriptional regulator involved in TMJC cartilage development. Collectively, our dataset serves as a valuable resource to decipher the cellular composition and regulatory signalling networks of TMJC development and enables molecular classification of various cell subtypes. This study not only advances our knowledge of human embryonic TMJ development, but also provides preliminary evidence for the potential regulatory function of *MXD4*, laying a foundation for further exploring the pathogenesis of congenital TMJ disorders induced by abnormal TMJC development.

## Materials and methods

### Collection of human embryonic TMJC

This study received ethical approval from the Institutional Review Boards of Nantong University Hospital (2020-K013). Informed consent was obtained from the parents of all participating individuals. One human embryonic TMJC sample was included for each gestational stage, including GW12, GW15, GW19, GW22, and GW25. For scRNA-seq, the TMJC tissue was surgically removed. The entire mandible, encompassing the mandibular body and the TMJ (including the condyle, joint capsule, joint disc, a section of the fibrous ligament, and the temporo-articular fossa), was isolated along the mandibular labiobuccal plane. Following this, the mandibular body, condyle, and coronoid process were detached from the remaining TMJ structure. The condyle was further separated from the mandibular body at its neck. The left condyle was carefully dissected and placed in a tissue preservation solution (Singleron Biotechnologies) before being transported under cold chain conditions for cell dissociation and subsequent single-cell sequencing.

### Preparation of single-cell suspensions

The dissociation process for the human embryonic TMJC was performed according to a previously established protocol.^2^ The isolated TMJC tissue was cut into 2 to 3 mm sections and rinsed with Hanks’ Balanced Salt Solution. Enzymatic digestion was performed for 15 minutes using a tissue dissociation solution (Singleron Biotechnologies). The resulting cell suspension was filtered through a sterile 40-μm filter and centrifuged at 150 g for 5 minutes. Erythrocytes were removed with a lysis reagent (Singleron Biotechnologies) for 10 minutes. The cells were resuspended in phosphate-buffered saline and stained with trypan blue (T6146, Sigma). Cell viability was assessed using a TC20 automated cell counter (Bio-Rad).

### Library preparation and data preprocessing

A cell suspension at a concentration of 1 × 10^5 cells/mL was prepared and loaded into a microfluidic plate. The scRNA-seq libraries were constructed using the Single-Cell RNA Library Kit (Singleron Biotechnologies). The prepared libraries were pooled and sequenced on an Illumina NovaSeq 6000 platform. Raw sequencing reads were processed with the CeleScope pipeline (https://github.com/singleron-RD/CeleScope). Initially, low-quality reads and adapter sequences were trimmed using FastQC (version 0.11.7) and Cutadapt (version 1.17). Reads were aligned to the GRCh38 genome (Ensembl V. 92 annotation) with STAR (version 020201). Gene and unique molecular identifier counts were generated using FeatureCounts (version 1.6.2). The CeleScope pipeline facilitated a series of analytical steps, leading to the construction of a gene expression matrix. The sequencing depth, represented by mean reads per cell, ranged from 34,174 to 53,672 across the five samples: 34,174 for GW12, 48,410 for GW15, 53,672 for GW19, 52,144 for GW22, and 49,563 for GW25.

### Dimension reduction and clustering analysis

For dimension reduction and cell clustering, the Seurat package (version 5) in R (https://satijalab.org/seurat/) was employed. To address batch effects and normalize expression data, the sctransform method was utilized (https://github.com/ChristophH/sctransform). The ‘subset’ function in Seurat was used to extract subsets of Seurat objects meeting specific quality control criteria (nFeature_RNA > 200 and nFeature_RNA < 6000 and nCount_RNA < 30,000 and percent.mt <40). Cells identified as low quality were filtered out and excluded from further analyses. A dedicated computational doublet-removal algorithm was not applied; however, cells with abnormally high gene counts or unique molecular identifier counts were excluded using the above quality-control thresholds to reduce the potential influence of multiplets. The top 2000 variable genes were chosen for sample data integration. Cell clusters were determined using the ‘FindNeighbors’ function across 50 dimensions, followed by the ‘FindClusters’ function with a resolution set to 0.2. This resolution was selected after comparing clustering patterns across multiple resolutions because it provided clear separation of major TMJC cell populations while avoiding excessive fragmentation of biologically related clusters at higher resolutions. For subclustering chondrocytes1 (Chondro1), a resolution of 0.2 was used. Clusters were visualized using the UMAP algorithm. Differentially expressed genes (DEGs) were identified using Seurat’s ‘FindMarkers’ function, with genes classified as DEGs if expressed in more than 25% of cells within their respective clusters and with a mean log₂ (Fold Change) greater than 0.25.

### Cell type annotation and gene enrichment analysis

Cell types were identified based on the expression profiles of well-established marker genes from the literature. Following this, gene enrichment analysis was performed using clusterProfiler v3.6.1 (https://bioconductor.org/packages/3.11/bioc/html/clusterProfiler.html). Biological processes (BP) with adjusted *P* value <.05 were considered significantly enriched. Gene Ontology (GO) classification was carried out using the org.Hs.eg.db database (https://bioconductor.org/packages/3.11/data/annotation/html/org.Hs.eg.db.html).

### Trajectory analysis

Pseudotime trajectories for the TMJC cell clusters were analysed using Monocle 3 (https://cole-trapnell-lab.github.io/monocle3). Highly variable genes from each cluster were selected, and trajectories were visualized using the UMAP method. Cells were then ordered according to their developmental and differentiation pathways. To identify genes dynamically regulated during differentiation, differential expression analysis along pseudotime was performed using the graph_test function in Monocle 3. Genes with q_value <0.01 and Moran’s *I* > 0.2 were considered significantly associated with differentiation. Gene Coexpression Module Identification and Functional Enrichment Analysis. To further dissect the regulatory mechanisms, pseudotime-dependent genes were clustered into coexpression modules using hierarchical clustering (Ward.D2 method, cutree_rows = 4). A pseudotime heatmap was generated (pheatmap package) to visualize dynamic expression patterns, with modules colour-coded to highlight distinct gene expression dynamics.

### Transcription factor analysis

Transcription factor analysis was conducted using the Python implementation of Single-Cell Regulatory Network Inference and Clustering (pySCENIC). Prior to analysis, the filtered expression matrix, formatted as a Seurat object, was converted to Loom format with the Seurat-disk R package. The pySCENIC pipeline involved four main steps: gene regulatory networks were inferred from coexpressed genes, candidate target genes were refined based on known transcription factor binding sites, area under the recovery curve scores were computed for the enriched gene list, and the area under the recovery curve matrix was subsequently binarized.

### Cell–cell communication analysis

Cell–cell communication networks based on ligand-receptor interactions were assessed with CellChat (http://www.cellchat.org/; last accessed on May 10, 2021). The functions ‘computeCommunProb’ and ‘aggregateNet’ in CellChat were used to quantify the number of ligand-receptor interactions and their intensity within the TMJC cell population. Overexpressed signalling genes and ligand–receptor interactions (pairs) were found by using ‘IdentifyOverExpressedGenes’ and ‘IdentifyOverExpressedInteractions’ within the CellChatDB for five datasets (GW12, GW15, GW19, GW22, and GW25).

### hdWGCNA analysis

High-dimensional weighted gene coexpression network analysis (hdWGCNA) is a method for analysing high-dimensional gene expression data, such as scRNA-seq, based on WGCNA. We conducted the analysis following the standard procedure using the hdWGCNA package (version 0.1.1.9010). First, the single-cell data were preprocessed using the Seurat package, selecting genes expressed in at least 5% of the cells as input for hdWGCNA analysis. To reduce data sparsity, the k-nearest neighbours algorithm was applied to cluster cells, and the average gene expression of each cluster was calculated to generate a metacell expression matrix. Next, the TestSoftPowers function was used to determine the optimal soft threshold for constructing the weighted gene coexpression network. Hierarchical clustering was then performed to identify gene modules. Subsequently, the module eigengenes were computed and normalized, and the intramodular connectivity (kME) of each gene was calculated to identify hub genes. To visualize module characteristics, colours were assigned to each module, and the clustering of gene modules and their spatial distribution were visualized using dendrograms and UMAP plots. Additionally, GO enrichment analysis was performed using the enrichR package to assess the BP enrichment among the hub genes of each module, and the results were visualized as enrichment plots.

### Zebrafish data acquisition

The scRNA-seq of zebrafish has been described by Saunders et al.^18^ The datasets were directly obtained from the NCBI Gene Expression Omnibus (GEO) repository under accession number GSE202639.

### Zebrafish strains and maintenance

Zebrafish were raised and maintained according to standard protocols previously described, with embryo staging based on hours postfertilization (hpf). Unless otherwise specified, embryos were kept at 28.5°C for all experiments. This study utilized the transgenic line *Tg (sox10: eGFP)*. To prevent pigmentation during in situ hybridization and imaging, 0.003% 1-phenyl-2-thiourea (PTU, Sigma P7629) was added to the E3 medium at 20 hpf. All animal procedures followed the guidelines approved by the Institutional Animal Care and Use Committee at Nantong University.

### Generation of zebrafish *mxd4* mutant lines using the CRISPR-Cas9 system

sgRNA targeting the *mxd4* gene was designed using the CRISPR design tool (https://www.crisprscan.org/?page=welcome). The target sequence was 5′-GGGGTGCTATCAGGACCCAA-3′. gRNA templates containing a T7 promoter were generated using the GenCRISPR sgRNA synthesis kit (GenScript Biotech Co., Ltd.), and gRNAs were synthesized by in vitro transcription. The pXT7-zCas9 plasmid was linearized using XbaI and then transcribed in vitro to generate Cas9 mRNA using the mMESSAGE mMACHINE T7 kit (Invitrogen, AM1344). At the one-cell stage, embryos were injected with a 2 to 3 nL solution containing 300 ng/µL Cas9 mRNA and 150 ng/µL sgRNA.

### mRNA synthesis and microinjection

*mxd4* DNA fragments were synthesized by PCR using the primers *mxd4*-BamHI-F and *mxd4*-EcoRI-R. The DNA fragments were subcloned into the pCS2+ vector (NOVOPRO, V011726), and the recombinant plasmid was linearized using the restriction endonuclease NotI (NEW ENGLAND, R0189V). The linearized product was purified as a template and transcribed into mRNA in vitro using the mMESSAGE mMACHINETM SP6 kit (Invitrogen, AM1340). Then the synthesized *mxd4* mRNA was coinjected with *mxd4*-sgRNA into one-cell-stage zebrafish embryos.

### Confocal imaging and statistical analysis

For confocal imaging, embryos were positioned laterally in 1.0 mL of 0.7% low-melting-point agarose (Invitrogen, 16520050) and covered with E3 medium. Imaging was conducted using a Nikon A1R confocal microscope. Z-stack images were acquired with a 3.0 μm step size under optimized imaging settings. Maximum intensity projections and slice projections were generated in NIS-Elements software, while 3D reconstructions and animations were created with NIS-Elements AR. All images were assembled in Adobe Photoshop, and diagrams were designed in Adobe Illustrator. The selection of craniofacial measurement parameters was based on previous studies and conducted using ImageJ software.^19^^,^^20^ Statistical analyses were conducted using GraphPad Prism v8. Data are presented as mean ± SEM. Comparisons among multiple groups were performed using one-way ANOVA followed by Tukey’s multiple comparisons test. *P* values <.05 were considered statistically significant. *, **, ***, and **** indicate *P* < .05, *P* < .01, *P* < .001, and *P* < .0001, respectively; ns indicates no significant difference.

### Zebrafish bulk RNA-seq analysis

Total RNA was isolated from zebrafish using the RNeasy mini kit (Qiagen no. 74126). After a sample quality check, the samples with the highest RIN (RNA integrity number) were selected for library preparation. Sequencing libraries were constructed using the Illumina TruSeq RNA Preparation Kit. High-throughput sequencing was performed using Illumina NovaSeq6000 with 100 bp pair-end according to the manufacturer’s instructions. Trim-galore was used to remove low-quality reads. Reads were aligned to the zebrafish reference genome using STAR (v.2.4.1d). HTSeq-0.6.1 was used for gene mapping. DEGs between experimental groups were identified using DESeq2 v.1.10.1.

## Results

### Cell type identification by scRNA-seq analysis of human embryonic TMJC

**Fig. 1:**
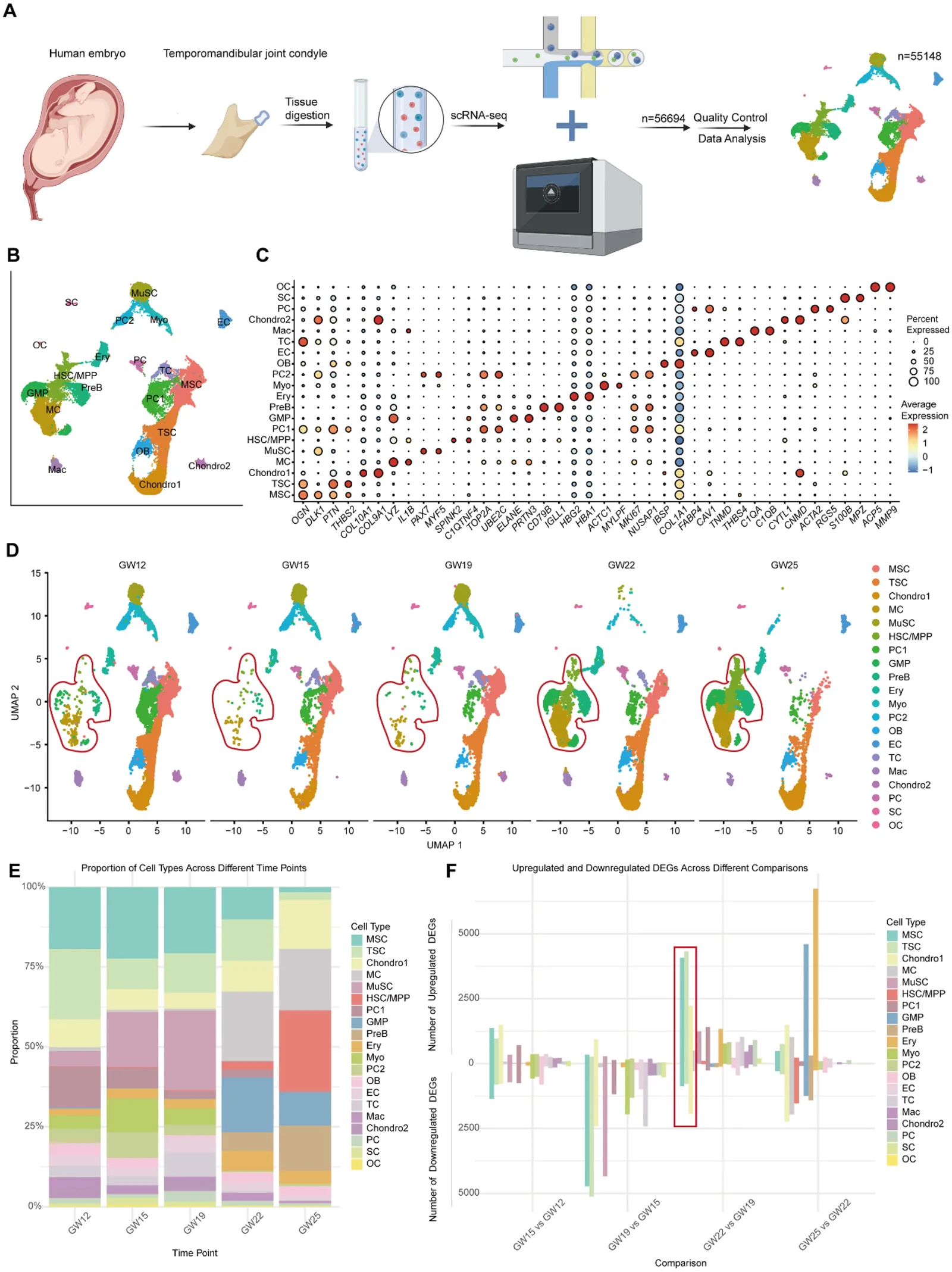
Cell type identification by scRNA-Seq analysis of human embryonic TMJC. (A) Schematic representation of human TMJC tissues dissected for single-cell transcriptomic analysis. (B) UMAP plot showing 20 major cell populations identified in the human TMJC using Seurat clustering at a resolution of 0.2. (C) The dot plot showing the expression profiles of marker genes used for major cell-type annotations. (D) UMAP plots showing the major cell types identified in human TMJC across different embryonic developmental stages. (E) Cell-type proportions across different developmental stages of the TMJC. (F) Number of upregulated and downregulated DEGs in each cell type between adjacent developmental stages.

We collected TMJC samples from five stages of human embryonic development (GW12, GW15, GW19, GW22, and GW25) and performed scRNA-seq on condylar cells (Figure 1A). A total of 56,694 cells were captured, and 55,148 high-quality cells were retained after quality control (Appendix Figure 1A), comprising 16,988 cells at GW12, 6796 at GW15, 8548 at GW19, 9787 at GW22, and 13,029 at GW25. Unsupervised clustering using the Seurat package identified 20 distinct cell populations (Figure 1B, C), including MSC, transitional state cells (TSC), Chondro1, chondrocytes2 (Chondro2), osteoblasts (OB), osteoclasts (OC), endothelial cells (EC), pericytes (PC), Schwann cells (SC), tenocytes (TC), myeloid cells (MC), macrophages (Mac), granulocyte–monocyte progenitors (GMP), hematopoietic stem/multipotent progenitor cells (HSC/MPP), preB cells (PreB), erythrocytes (Ery), muscle satellite cells (MuSC), myocytes (Myo), and two proliferating cell populations (PC1 and PC2). A heatmap showing the top five DEGs for each cell population is presented in Appendix Figure 1B, and GO enrichment analyses for all clusters are shown in Appendix Figure 1C.

Analysis of cell numbers and proportions across developmental stages revealed pronounced temporal changes in TMJC cellular composition (Figure 1D, E; Appendix Figure 2A, B). Notably, immune cell populations were scarce at GW12, GW15, and GW19, but increased markedly at GW22 and GW25, indicating that immune cells emerge in the TMJC between GW19 and GW22.

To further investigate stage-specific transcriptional changes during condylar development, we performed differential gene expression analysis between adjacent developmental stages. Among all cell populations, MSC, TSC, and Chondro1 exhibited the highest numbers of upregulated DEGs between GW19 and GW22 (Figure 1F), suggesting that this interval represents a critical developmental window for TMJC remodelling.

GO enrichment analysis revealed that genes upregulated in MSC at GW22 compared with GW19 were primarily enriched in ossification, extracellular matrix organization, and extracellular structure organization, indicating an active role of MSC in cartilage and bone formation during this stage of TMJC development. In contrast, upregulated genes in Chondro1 were significantly enriched in oxidative phosphorylation and apoptotic signalling pathways (Appendix Figure 2C), suggesting that these cells may be undergoing hypertrophic changes, a process closely associated with endochondral ossification.

### Trajectory analysis reveals a mesenchymal-to-chondrocyte differentiation program during TMJC development

**Fig. 2:**
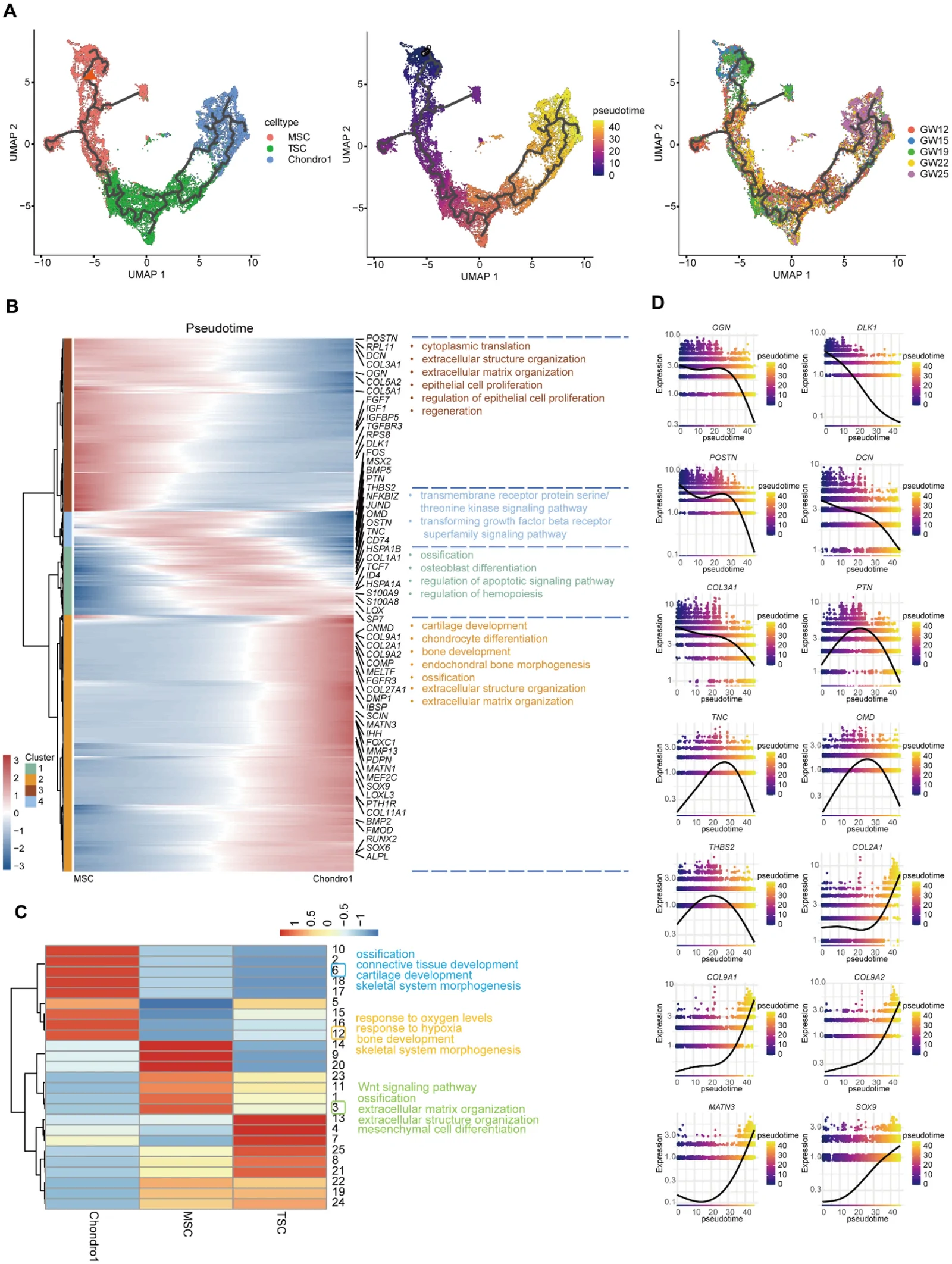
MSC differentiation into Chondro1 in human embryonic TMJC. (A) UMAP plots showing the pseudotime trajectory from MSC to Chondro1. (B) Heatmap showing dynamic gene expression changes along the MSC-to-Chondro1 trajectory. (C) Gene module analysis showing module enrichment during MSC-to-Chondro1 differentiation. Colour bars indicate module scores. (D) Pseudotime kinetics showing changes in MSC, TSC, and Chondro1 marker gene expression along the differentiation trajectory.

To clarify TMJC cell differentiation, we analysed 20 cell clusters and identified three major developmental trajectories, including a mesenchymal–skeletal lineage, a hematopoietic lineage, and a muscle-associated lineage (Appendix Figure 3A). Notably, the Chondro1 cluster exhibited high expression of *COL10A1*, a well-established marker of chondrocyte hypertrophy (Figure 1C, D), indicating that this population represents a hypertrophic chondrocyte-like state associated with endochondral ossification. We focused on the mesenchymal-to-chondrocyte trajectory for subsequent analyses. Pseudotime reconstruction positioned MSC upstream of Chondro1 and revealed a continuous differentiation trajectory, which was further supported by the gestational-age distribution of TMJC samples along pseudotime (Figure 2A and Appendix Figure 3B).

Gene expression dynamics along the MSC → Chondro1 trajectory revealed a stepwise transcriptional transition. Early-stage genes were enriched for biosynthetic and extracellular matrix organization processes, intermediate-stage genes were associated with TGF-β receptor signalling, and late-stage genes were associated with cartilage development and endochondral ossification (Figure 2B). Module analysis yielded consistent results, with Module 6 highly expressed in Chondro1 and mainly enriched for ossification and cartilage development (Figure 2C). Consistent with lineage progression, MSC markers gradually decreased, TSC markers showed a transient increase, and chondrocyte markers progressively increased (Figure 2D).

To identify key gene regulatory modules underlying TMJC chondrogenesis, we performed hdWGCNA and identified 17 coexpression modules (Appendix Figure 4A, B). Among these, the chondrocyte-enriched module M6 was strongly associated with skeletal system development and cartilage-related processes (Appendix Figure 4C, D). Hub-gene analysis highlighted established cartilage regulators, including *SOX9, COL9A1*, and *COL9A2*, as well as less-characterized genes such as *FGFBP2, SGSM2*, and *ITIH6*. These candidate genes were progressively upregulated along MSC → Chondro1 differentiation (Appendix Figure 4E, F), suggesting that they may be involved in condylar cartilage development.

### Signal pathway in chondrocyte cell development

**Fig. 3:**
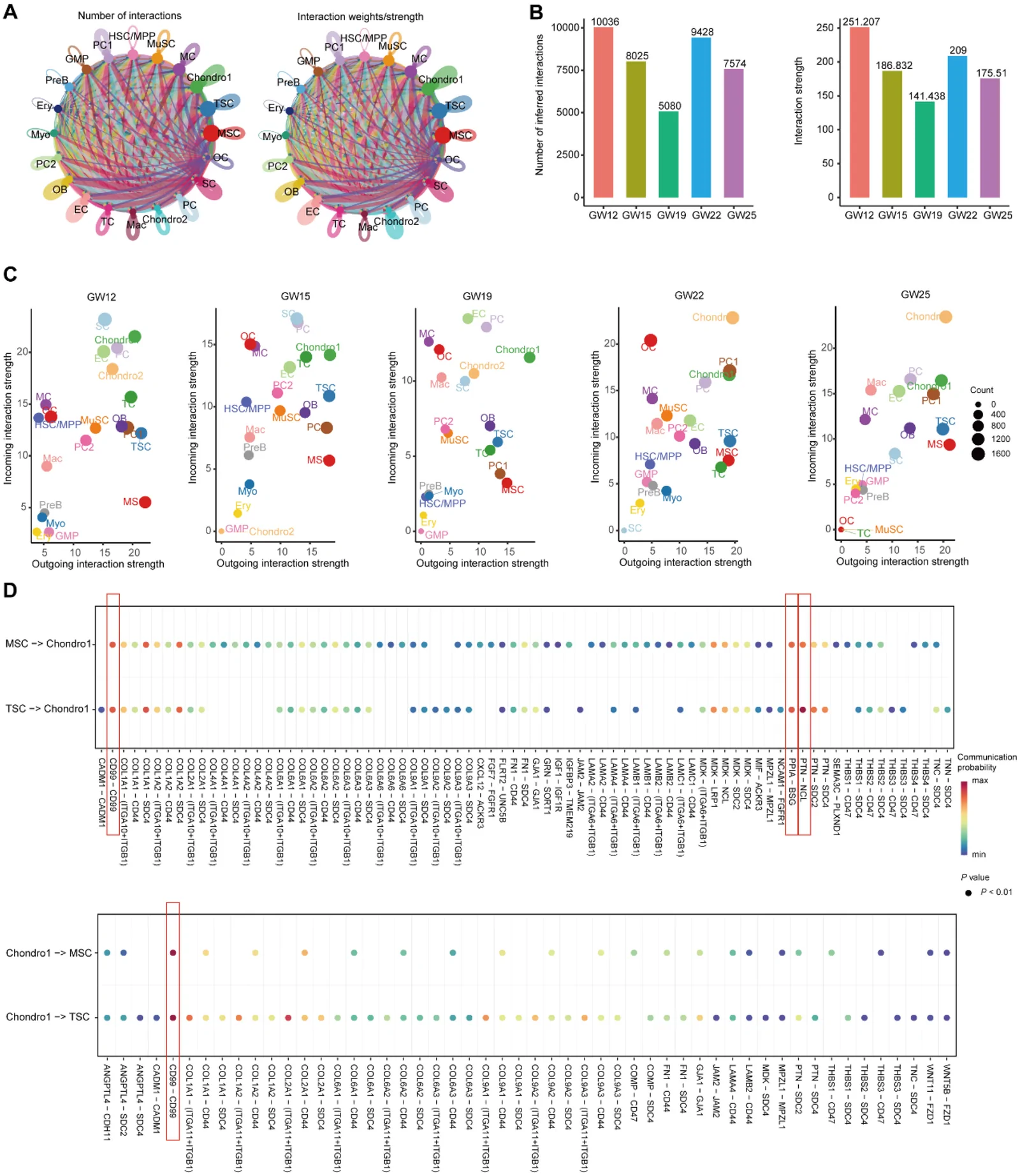
Cell–cell communication dynamics during TMJC development. (A) The number and interaction strength of signalling pathways in 20 cell clusters. (B) Bar charts showing the number and overall strength of intercellular interactions across developmental stages. (C) Scatter plots showing incoming and outgoing signalling patterns of major cell populations at different developmental stages.(D) Analysis of key ligand–receptor interactions among MSC, TSC, and Chondro1.

To dissect how intercellular interactions within the TMJC microenvironment regulate signalling pathways, we performed a systematic analysis of cell–cell communication across distinct cell populations. We first evaluated the total number of interactions and interaction strengths among different cell types within the TMJC (Figure 3A, B). The results revealed marked differences in the extent of intercellular communication across developmental stages, indicating dynamic remodelling of signalling interactions during TMJC development.

We next compared the incoming and outgoing interaction strengths of individual cell populations, with a particular focus on the Chondro1 cluster (Figure 3C). Notably, Chondro1 exhibited relatively high levels of both incoming and outgoing signalling across multiple developmental stages, suggesting that it functions not only as a major signal-receiving population but also as an important signalling sender. These findings position Chondro1 as a central hub within the TMJC cell–cell communication network.

We further interrogated ligand–receptor signalling pathways among MSC, TSC, and Chondro1, with an emphasis on signals received by and emitted from Chondro1 (Figure 3D). Among the signalling pathways received by Chondro1, CD99–CD99, PPIA–BSG, and PTN–NCL exhibited high communication probabilities, indicating potential roles in regulating Chondro1 cell state and function. In parallel, analysis of signalling pathways transmitted from Chondro1 to MSC and TSC revealed that CD99–CD99 was also prominently enriched, suggesting its involvement in bidirectional communication among MSC, TSC, and Chondro1.

In addition, we examined the temporal dynamics of these key ligand–receptor interactions across developmental stages (Appendix Figure 5A). CD99–CD99 signalling showed consistently high communication probabilities among MSC, TSC, and Chondro1 across all examined stages, suggesting that it may serve as a stable and fundamental signalling axis during TMJC development. In contrast, PTN–NCL signalling increased progressively over developmental time, implying a more prominent role in later stages of TMJC development. Conversely, PPIA–BSG signalling was more pronounced at early stages and gradually diminished at later stages, suggesting that it may primarily participate in early intercellular communication events.

### Transcription factor analysis in TMJC development

**Fig. 4:**
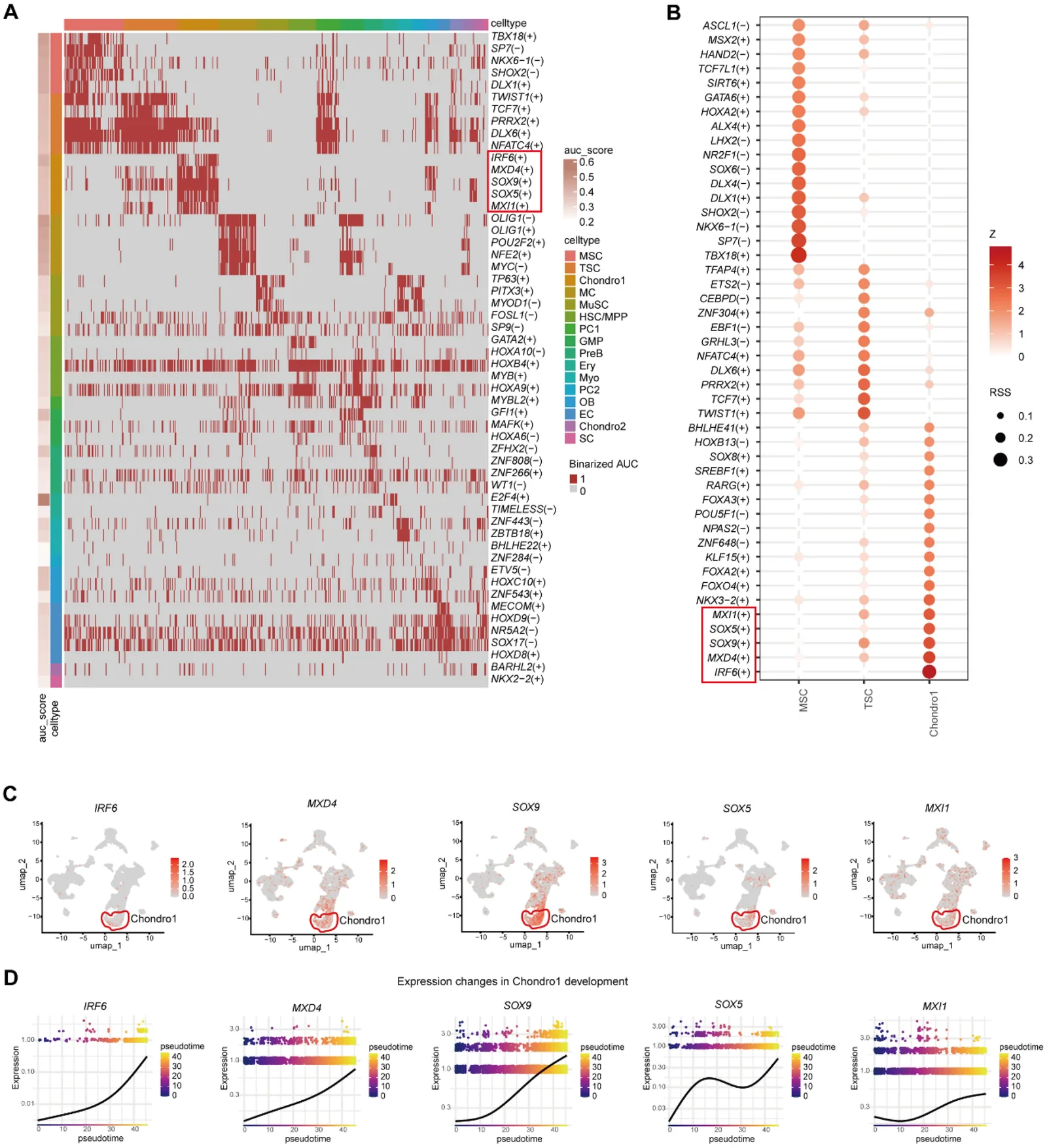
The analysis of transcription factors during the development of TMJC (A) Binarized heatmap showing cluster-specific activated regulons. (B) Cell cluster-specific transcription factor activity analysis based on *Z*-scores and RSS values. The dot size represents RSS, while the colour intensity indicates *Z*-score levels. (C) UMAP plots displaying the expression patterns of *IRF6, MXD4, SOX9, SOX5,* and *MXI1* across different cell clusters. (D) The pseudotime trajectory from MSC to Chondro1, showing the expression dynamics of *IRF6, MXD4, SOX9, SOX5*, and *MXI1* along the pseudotime.

Using SCENIC, we identified changes in the gene regulatory networks across TMJC cell populations. Binarized heatmap shows that *IRF6, MXD4, SOX9, SOX5*, and *MXI1* are turned on in Chondro1. Furthermore, the most active transcription factors in Chondro1 were also *IRF6, MXD4, SOX9, SOX5*, and *MXI1* (Figure 4A, B).

Chondro1 represents the process of endochondral osteogenesis within the TMJC, a critical biological mechanism in the development of the TMJ and mandible. Our further analysis demonstrated that *MXD4, SOX9*, and *SOX5* are particularly highly expressed in Chondro1, suggesting that they play crucial roles in maintaining chondrocyte identity (Figure 4C). Pseudotime analysis revealed that these transcription factors are progressively upregulated across different developmental stages, reaching peak expression in Chondro1 cells (Figure 4D). This indicates that these transcription factors may be involved in the regulation of endochondral osteogenesis.

### Loss of *mxd4* reduces zebrafish craniofacial size

**Fig. 5:**
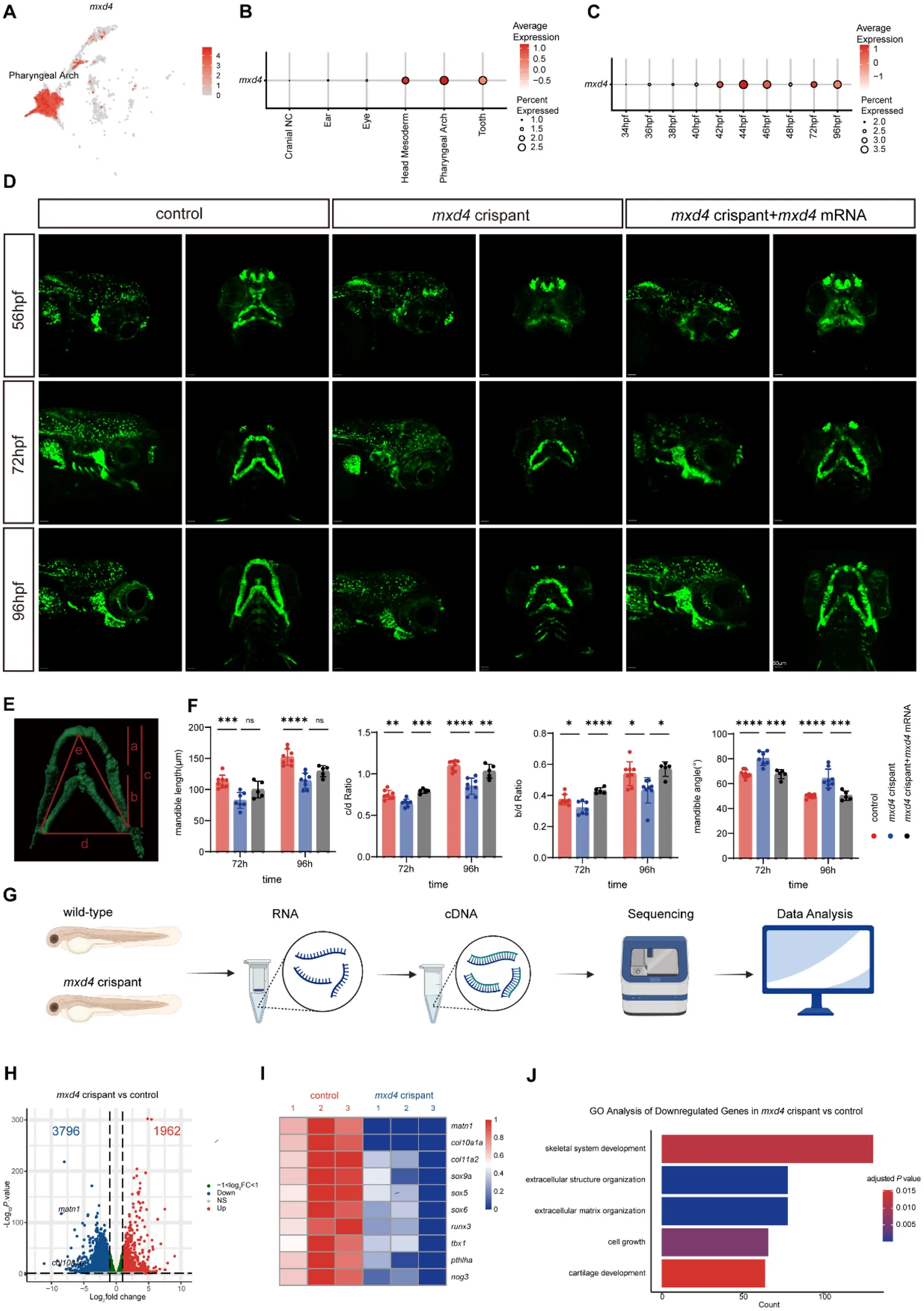
Functional analysis of *mxd4* in zebrafish craniofacial development. (A) UMAP plots displaying the expression of *mxd4* in zebrafish. (B) The dot plot showing *mxd4* expression across different zebrafish tissues. (C) The dot plot showing *mxd4* expression in the pharyngeal arches at different developmental stages. (D) Representative images of jaw development at 56, 72, and 96 hpf in control, *mxd4* crispant, and *mxd4* crispant + *mxd4* mRNA rescue groups. Scale bar, 50 μm. (E) Schematic diagram of jaw measurement parameters. (F) Quantification of jaw measurement parameters. Line a represents the mandible length, while Line d serves as the baseline for subsequent measurements. Line b indicates the distance from Line d to the anterior end of the ceratohyal, and Line c represents the distance from Line d to the anterior end of Meckel’s cartilage. The ratios b/d and c/d were quantified. Additionally, Angle e represents the angle of Meckel’s cartilage. (G) Workflow diagram of RNA Sequencing for wild-type and *mxd4* crispant zebrafish. (H) Volcano plot showing differentially expressed genes in *mxd4* crispants compared with controls. (I) Heatmap showing differentially expressed cartilage-related genes between *mxd4* crispant and control groups. (J) GO enrichment analysis of downregulated genes in *mxd4* crispants relative to controls.

We selected *MXD4* for further functional investigation. Sequence alignment between zebrafish and human MXD4 proteins revealed a 96.23% similarity in helix-loop-helix domain (Appendix Figure 6A). The zebrafish pharyngeal arch will eventually differentiate into the maxilla, mandible, and jaw joint. We also analysed *mxd4* expression in the zebrafish pharyngeal arch. The results showed that *mxd4* is expressed in the zebrafish pharyngeal arch, with an increasing trend in expression from 34 to 96 hpf (Figure 5A-C). To validate the efficiency of the G0 crispant strategy, we performed Sanger sequencing of the targeted region after sgRNA injection. The sequencing results confirmed the presence of mutations at the *mxd4* target site in G0 embryos, supporting the successful generation of *mxd4* mutants (Appendix Figure 6B). In zebrafish, *mxd4* mutation leads to mandibular hypoplasia, which can be partially rescued by *mxd4* mRNA (Figure 5D-F). To gain detailed insights into the molecular and genetic changes caused by *mxd4* disruption, RNA sequencing was performed (Figure 5G). Gene expression profiles were compared between *mxd4*-crispant and wild-type embryos at 56 hpf across three independent experiments. The results revealed that mxd4 disruption led to significant changes in gene expression, with 3796 genes downregulated and 1962 genes upregulated. Among the cartilage-related genes, *matn1* and *col10a1* showed the most pronounced downregulation in the *mxd4*-crispant group (Figure 5H, I). Additionally, GO analysis revealed that the downregulated genes were enriched in BP related to cartilage development and skeletal system development (Figure 5J; Appendix Figure 6C). Motif prediction analysis using the JASPAR database identified putative MXD4 binding sites in the regulatory regions of human *MATN1* and *COL10A1*, suggesting a potential regulatory role in cartilage-related gene expression (Appendix Figure 6D). Furthermore, pseudotime trajectory analysis during the differentiation of MSC into Chondro1 populations in human TMJC revealed consistent dynamic expression patterns of *MXD4, MATN1*, and *COL10A1* (Appendix Figure 6E, F).

## Discussion

In this study, we refined the single-cell atlas of the human TMJC across five embryonic developmental stages, providing a comprehensive view of its cellular composition and developmental dynamics. Our analysis delineates the complex and heterogeneous cellular landscape of this unique bilateral hinge joint, identifying 20 major cell types and highlighting the remarkable cellular diversity underlying TMJC development. Compared with our previous single-cell analysis of human embryonic condyles at 3 to 4 months of gestation, the present study further expanded the developmental time window and captured stage-dependent cellular and regulatory changes that were not apparent in the earlier dataset. The expanded atlas further revealed the stage-specific emergence of immune cells, the dynamic differentiation process from MSC to Chondro1 cells, hub genes associated with condylar chondrocytes, and the potential role of *MXD4* in regulating condylar chondrogenesis, thereby providing new insights into the spatiotemporal dynamics of cell fate transitions and molecular regulation during TMJC development.

Previous studies have reported that condylar cartilage tends to exhibit distinct morphological features by approximately GW15, followed by rapid endochondral ossification and formation of the bone marrow cavity by GW17.^21^ Additional studies have shown that hematopoietic tissue begins to appear in the condyle during the maturation period (after GW20).^2^^,^^5^ In the present study, we further characterized the temporal dynamics of this process at the single-cell level. Our results showed that immune cells were scarcely detectable at GW19 but increased markedly by GW22. This finding is consistent with the established timeline of bone marrow development and further identifies the GW19–GW22 window as a critical phase for the establishment of the immune cell niche within the condylar marrow microenvironment, thereby complementing and refining the relevant developmental chronology.

Condylar growth and development are indispensable for mandibular morphogenesis and TMJ formation, which rely on highly coordinated processes of tissue patterning and cell fate determination.^22^, ^23^, ^24^ Our prior single-cell profiling of human embryonic condyles at 3 to 4 months of gestation demonstrated that condylar cartilage originates from primitive MSC.^2^ Building on this foundation, the present study incorporates specimens spanning successive developmental stages and captures the spatiotemporal dynamics of gene expression. This expanded developmental window provides temporal context for MSC-to-Chondro1 differentiation, showing that this process is accompanied by stage-specific changes in cellular composition and transcriptional programs during embryonic development. In addition to intrinsic transcriptional changes, our cell–cell communication analysis revealed dynamic remodelling of intercellular signalling during TMJC development. Chondro1 exhibited relatively high levels of both incoming and outgoing signalling interactions, suggesting that they may serve as a central communication hub during condylar chondrogenesis. Ligand–receptor pathways such as CD99–CD99, PPIA–BSG, and PTN–NCL were found to be involved in regulating signal reception and transmission by Chondro1 cells. Moreover, these three pathways displayed distinct spatiotemporal expression patterns across developmental stages, collectively revealing the complex mechanisms by which stage-specific signals co-ordinately regulate TMJ chondrogenesis and development.

Condylar cartilage functions as a key growth centre in craniofacial development, driving mandibular elongation and shape remodelling through endochondral ossification.^25^^,^^26^ This process encompasses sequential stages of chondrocyte proliferation, differentiation, hypertrophy, and eventual replacement by bone, and disruption of any of these steps can result in mandibular developmental abnormalities.^11^ In our single-cell atlas, the Chondro1 population displayed transcriptional features characteristic of an endochondral ossification–associated chondrocyte state, supporting its involvement in condylar cartilage maturation. Regulatory network analysis identified several transcription factors associated with the Chondro1 state, including *IRF6, MXD4, SOX9, SOX5*, and *MXI1*. The recovery of known chondrogenic regulators such as *SOX9* and *SOX5* supports the validity of this analysis,^27^^,^^28^ while the less-characterized factors *IRF6, MXD4*, and *MXI1* may provide new candidates for investigating condylar cartilage development. Previous studies have demonstrated that *IRF6* plays a critical role in cranial neural crest development, with *IRF6* mutations associated with craniofacial malformations.^29^^,^^30^ MXD4 and MXI1 belong to the MAD family of transcription factors, which are known to regulate fundamental cellular processes such as proliferation, differentiation, and apoptosis,^31^^,^^32^ but their functions in cartilage biology have not been explored.

Based on its expression pattern in Chondro1 and along the cartilage differentiation trajectory, we selected *MXD4* for further investigation. Loss-of-function experiments in zebrafish revealed that *mxd4* deficiency resulted in mandibular underdevelopment. This phenotype may be associated with altered jaw joint development, which could indirectly affect mandibular skeletal growth. Transcriptomic analysis further showed that *mxd4* functional deficiency significantly affected cartilage- and skeletal-development–related pathways, accompanied by marked downregulation of *matn1* and *col10a1*. MATN1 is an extracellular matrix protein involved in cartilage structure, while COL10A1 is a marker of hypertrophic chondrocytes required for terminal differentiation during endochondral ossification.^33^, ^34^, ^35^, ^36^ The reduced expression of *matn1* and *col10a1* in *mxd4*-deficient models suggests that these genes may represent downstream effectors associated with *mxd4*-dependent developmental regulation. Meanwhile, analysis of our human TMJC single-cell transcriptomic dataset revealed that *MXD4, MATN1*, and *COL10A1* exhibited consistent expression dynamics along the MSC-to-Chondro1 differentiation trajectory, providing further cross-species support for the potential involvement of *MXD4* in cartilage development. Notably, motif prediction analysis identified putative MXD4 binding sites within the regulatory regions of *MATN1* and *COL10A1*, suggesting a potential regulatory relationship. However, whether MXD4 directly regulates *MATN1* and *COL10A1* transcription, or instead acts through indirect regulatory networks, remains to be fully determined. Nevertheless, these predictions require further experimental validation to confirm direct transcriptional regulation. Likewise, the upstream signals that activate *MXD4* during the MSC-to-Chondro1 transition require further investigation.

Importantly, our findings may also have clinical relevance for human TMJ disorders. Abnormal condylar cartilage development and defective endochondral ossification are closely associated with various craniofacial and TMJ-related disorders, including condylar hypoplasia, mandibular deficiency, and TMJ osteoarthritis.^37^^,^^38^ In this study, *MXD4* was identified as a candidate regulator of condylar chondrogenesis. Theoretically, dysregulated *MXD4* expression may affect endochondral ossification, ultimately leading to abnormal condylar growth and craniofacial skeletal malformations. Although direct evidence linking *MXD4* to human TMJ disorders remains lacking, *MXD4* and its downstream pathways may serve as potential molecular markers for evaluating joint developmental status and disease-related cartilage remodelling. From a translational perspective, this developmental atlas may help identify stage-specific biomarkers and prioritize molecular targets, thereby providing a reference for regenerative therapeutic strategies for TMJ disorders. In the future, targeted modulation of key regulatory pathways, including MXD4-associated signalling networks, may offer new therapeutic directions for condylar cartilage repair and regeneration.

However, due to the developmental differences between the zebrafish jaw joint and the human TMJ, this study has inherent limitations. The mammalian TMJ is a secondary joint, and its condylar cartilage represents secondary cartilage, with characteristics that differ fundamentally from those of the zebrafish jaw joint, which is a primary joint formed between the posterior Meckel’s cartilage and anterior palatoquadrate.^39^^,^^40^ Therefore, the developmental mechanisms and molecular signalling programs governing Meckel’s cartilage remodelling in zebrafish are not fully identical to those regulating endochondral ossification of the mammalian TMJ condyle. Nevertheless, the zebrafish model remains suitable for rapidly exploring the functions of *mxd4* in craniofacial cartilage and mandible development. Further investigations using mammalian models and human TMJ tissues are required to clarify the specific roles of *MXD4* in condylar secondary cartilage development and the pathogenesis of TMJ-related disorders.

In summary, this study established a single-cell-resolution developmental atlas of human embryonic TMJ cartilage, systematically analysing its cellular composition, differentiation trajectories, and core developmental characteristics. It further enriched previous research findings and clarified the temporal dynamic patterns of cells during development. Combined with in vivo functional experiments, we confirmed that the transcription factor *MXD4* serves as a candidate regulatory molecule involved in regulating condylar endochondral ossification. The present findings not only provide solid theoretical evidence for elucidating the developmental rules of TMJ cartilage and exploring the pathogenesis of related disorders, but also lay a data foundation and offer feasible research directions for subsequent molecular mechanism investigation and clinical translational research.

## Data availability

The datasets generated and analysed during the current study have been deposited at the NCBI Gene Expression Omnibus (GEO) repository: GSE290860.

## Funding

This work was supported by grants from the National Key R&D Program of China (2023YFC2509100), the Science and Technology Commission of Shanghai Municipality (23Y31900400), the Shanghai’s Top Priority Research Center (2022ZZ01017), National Natural Science Foundation of China (82370984), Dominant disease biological sample project of the Ninth People’s Hospital Affiliated to Shanghai Jiao Tong University School of Medicine (YBKA202201), Science and Technology Commission of Shanghai Municipality (22S31903400), the Natural Science Foundation of Shanghai (22ZR1437600), the Postgraduate Research & Practice Innovation Program of Jiangsu Province (KYCX25_3846).

## Author contributions

*Contributed to the conception and design of the study, data acquisition, analysis and interpretation, and drafted and critically revised the manuscript:* Wang. *Contributed to data analysis and critically revised the manuscript:* Chen. *Contributed to the study design and critically revised the manuscript:* Zhang. *Contributed to data acquisition and critically revised the manuscript:* Zhao, Xu, and Liu. *Contributed to the conception and design and critically revised the manuscript*: Zheng. *Contributed to the conception and design, data acquisition, and drafted and critically revised the manuscript*: Liu and Feng. *Contributed to drafting or critically revising the manuscript, approved the final version to be submitted, and agree to be accountable for all aspects of the work*: All authors.

## Conflict of interest

The authors declare that they have no known competing financial interests or personal relationships that could have appeared to influence the work reported in this article.

## Appendix Supplementary materials

Image, application 1
