# Supplementary material for: Single-Cell Atlas of Human Temporomandibular Joint Condyle Identifies MXD4 as a Regulator of Chondrogenesis
Source: Int Dent J. 2026 Sep 3;76(5):111096. doi: 10.1016/j.identj.2026.111096 (PMC13571596; doi:10.1016/j.identj.2026.111096)
Supplement: Supplementary file 1 [file mmc1.docx]

**Appendix Figures**

**Appendix Figure 1. Marker gene expression in cell types during the development of human embryonic TMJC**

(A) Scatter plots showing quality control metrics across GW12, GW15, GW19, GW22, and GW25 samples.
(B) Heatmap depicting the top 5 DEGs for each identified cell population.
(C) GO analysis of cell-type-specific genes for each cell population.

**Appendix Figure 2. Temporal dynamics of cell type proportions during human embryonic TMJC development**

**(A) UMAP plots showing the distribution of immune cells (highlighted in red) across different developmental stages of human embryonic TMJC. Other cell types are shown in gray for reference.**

**(B) Bar graphs illustrating the proportions of various cell types at different developmental stages.**

**(C)** GO analysis results showing the enrichment of upregulated genes in MSC and Chondro1 in GW22 vs GW19.

**Appendix Figure 3. Developmental distribution analysis of human embryonic TMJC cell types in pseudotime**

(A) Monocle3 showing the distribution of different cell types in human embryonic TMJC along the overall pseudotime trajectory.
(B) UMAP plot based on developmental time points, displaying the distribution of TMJC cells from various developmental stages (GW12, GW15, GW19, GW22, GW25) along the pseudotime trajectory.

**Appendix Figure 4. Hub gene analysis of chondrocyte Cells via WGCNA**

(A) Dendrogram of hierarchical clustering for module-specific genes during condylar development.

(B) UMAP plots showing the expression distributions of hub genes from each module across TMJC cell clusters.

(C) The dot plot showing the relationship between each module and cell populations.

(D) GO analysis revealing the main biological functions of M2, 4, 6.

(E) The top 25 hub genes of Module 6

(F) The expression dynamics of *FGFBP2*, *SGMS2* and *ITIH6* along the pseudotime trajectory.

**Appendix Figure 5. Temporal dynamics of Chondro1-associated ligand–receptor signaling pathways during TMJC development**

(A) CellChat-inferred communication patterns of Chondro1-associated ligand–receptor signaling pathways across different developmental stages of the TMJC (GW12, GW15, GW19, GW22, and GW25).

**Appendix Figure 6. Functional analysis of MXD4**

(A) Sequence alignment of human and zebrafish MXD4 showing 96.23% similarity within the conserved HLH domain.

**(B) Schematic of the zebrafish *mxd4* CRISPR/Cas9 target site and validation of target-site mutations by Sanger sequencing.**

(C) The chord diagram illustrating the enriched genes from the GO analysis of *mxd4* crispant compared to the control group, belonging to cartilage development (red) and skeletal system development (blue).

(D) Predicted MXD4-binding motifs and putative binding sites in the regulatory regions of human *MATN1* and *COL10A1*.

(E) Gene expression dynamics of *MXD4*, *MATN1*, and *COL10A1* along the pseudotime trajectory during the differentiation process from MSC to Chondro1 populations.

(F) The pseudotime trajectory from MSC to Chondro1, showing the expression dynamics of *MXD4*, *MATN1*, and *COL10A1* along the pseudotime.
